# Development of Physiologically Based Pharmacokinetic Model for Orally Administered Fexuprazan in Humans

**DOI:** 10.3390/pharmaceutics13060813

**Published:** 2021-05-29

**Authors:** Yoo-Seong Jeong, Min-Soo Kim, Nora Lee, Areum Lee, Yoon-Jee Chae, Suk-Jae Chung, Kyeong-Ryoon Lee

**Affiliations:** 1 College of Pharmacy and Research Institute of Pharmaceutical Sciences, Seoul National University, Seoul 08826, Korea; jus2401@snu.ac.kr (Y.-S.J.); misol@snu.ac.kr (M.-S.K.); 2 Daewoong Pharmaceutical Co., Ltd., Seoul 06170, Korea; nora37@gmail.com (N.L.); areum9893@daewoong.co.kr (A.L.); 3 College of Pharmacy, Woosuk University, Wanju-gun 55338, Korea; yjchae@woosuk.ac.kr; 4 Laboratory Animal Resource Center, Korea Research Institute of Bioscience and Biotechnology, Cheongju 28116, Korea

**Keywords:** DWP14012, fexuprazan, human scaling, physiologically based pharmacokinetic modeling, potassium-competitive acid blocker

## Abstract

Fexuprazan is a new drug candidate in the potassium-competitive acid blocker (P-CAB) family. As proton pump inhibitors (PPIs), P-CABs inhibit gastric acid secretion and can be used to treat gastric acid-related disorders such as gastroesophageal reflux disease (GERD). Physiologically based pharmacokinetic (PBPK) models predict drug interactions as pharmacokinetic profiles in biological matrices can be mechanistically simulated. Here, we propose an optimized and validated PBPK model for fexuprazan by integrating in vitro, in vivo, and in silico data. The extent of fexuprazan tissue distribution in humans was predicted using tissue-to-plasma partition coefficients in rats and the allometric relationships of fexuprazan distribution volumes (*V_SS_*) among preclinical species. Urinary fexuprazan excretion was minimal (0.29–2.02%), and this drug was eliminated primarily by the liver and metabolite formation. The fraction absorbed (*Fa*) of 0.761, estimated from the PBPK modeling, was consistent with the physicochemical properties of fexuprazan, including its in vitro solubility and permeability. The predicted oral bioavailability of fexuprazan (38.4–38.6%) was within the range of the preclinical datasets. The C_max_, AUC_last_, and time-concentration profiles predicted by the PBPK model established by the learning set were accurately predicted for the validation sets.

## 1. Introduction

Potassium-competitive acid blockers (P-CAB) are novel H^+^/K^+^ ATPase inhibitors administered for the treatment of gastric acid-related disorders including gastroesophageal reflux disease (GERD), gastric ulcer, and *Helicobacter pylori* (*H. pylori*) infection [1,2]. GERD is a common gastrointestinal disorder in the United States [3] and South Korea [4]. It is associated with serious complications such as Barrett’s esophagus and esophageal adenocarcinoma [3,4,5]. Several models have been constructed to elucidate the mechanism of GERD progression, and continuous esophageal stimulation by gastric acid is considered a major cause [3,4]. There is widespread use of gastric acid-neutralizing drugs, such as proton pump inhibitors (PPIs), to treat various gastric disorders [4,5]. However, PPIs have drawbacks related to their pharmacokinetics (short elimination half-life) and pharmacodynamics (slow onset of action and inability to control nocturnal acid secretion) [6,7,8]. While PPIs covalently bind the proton pump [7,8,9], P-CABs competitively and reversibly inhibit the potassium site of H^+^/K^+^ATPase and have relatively long plasma half-lives, which could lead to their rapid onset, long duration of action, and acid suppression efficacy being at least comparable to that of PPIs [7,9]. Hence, several P-CABs have already been approved, including revaprazan in South Korea (2005), vonoprazan in Japan (2015), and tegoprazan in South Korea (2018) [5]. The foregoing drugs constitute the next generation of PPIs [5].

Fexuprazan (DWP14012) is a new candidate P-CAB currently undergoing Phase 3 clinical trials on patients with erosive esophagitis (Daewoong Pharmaceutical, Co. Ltd., Seoul, Korea). A Phase 1 clinical study [10] revealed that fexuprazan has favorable kinetics, including rapid absorption (median T_max_: 1.75–3.5 h) and long elimination half-life (~9 h). Its inhibitory effect of gastric acid secretion was reached at ~2 h after the first 80–320-mg dose (pH ≥ 4.0), and this onset was significantly faster than that of the PPI esomeprazole (e.g., ~4 h). In addition, the duration of fexuprazan action was also maintained during the night, and the mean percentage of time that intragastric pH was above 4 was reasonably described in relation to the fexuprazan exposure in the plasma (AUC_tau_) [1]. Despite clinical evidence for its efficacy, it is nonetheless necessary to understand fexuprazan pharmacokinetics based on in vitro experiments showing its biotransformation by CYP3A4 [10], which might result in drug-drug interactions (DDIs) because acid suppression therapy is frequently co-administered with other drugs [11,12,13,14]. Physiologically based pharmacokinetic (PBPK) models are more useful for predicting potential DDIs than conventional compartmental approaches [14,15]. As PBPK models incorporate physiological and anatomical variables in their structure, these models may be rationally scaled to predict drug pharmacokinetics in various species (e.g., experimental animals to humans) and populations (e.g., children, the elderly, and individuals taking multiple medications). Preclinical studies disclosed differences in absolute fexuprazan bioavailability among rats, dogs, and monkeys (range: 3.89–50.6%) [16], despite its sufficient solubility (i.e., freely soluble at pH 4.0 and slightly water-soluble at pH 1.2 and 6.8 [16]) and permeability (e.g., comparable with highly permeable propranolol [17,18,19]). Its efflux ratio in Caco-2 systems was <2, and it was unaffected by MDR1, MRP, and BCRP inhibitors such as cyclosporin A, MK571, and fumitremorgin C [16]. Considering that absolute fexuprazan bioavailability data were unavailable for humans as clinical trials did not include intravenous fexuprazan pharmacokinetics, modeling with in vitro and in vivo PBPK parameters could help elucidate clinical fexuprazan pharmacokinetics.

In the present study, a PBPK model was developed for fexuprazan orally administered to humans at clinically relevant dosages using in vitro and in vivo experimental data and published data including metabolite formation kinetics and plasma protein binding [20,21]. We used pharmacokinetic data for experimental animals to determine the allometric relationship between volume of distribution and body weight and estimate fexuprazan tissue-to-plasma partition coefficients in humans. The proposed fexuprazan PBPK model was validated using separate clinical datasets [1,10,22,23]. It could also be expanded to predict fexuprazan pharmacokinetics in various clinical settings such as specific populations, which might be difficult to perform in clinical trials such as senior citizens or children, and in combination with other drugs.

## 2. Materials and Methods

### 2.1. Model Structure

**Figure 1:**
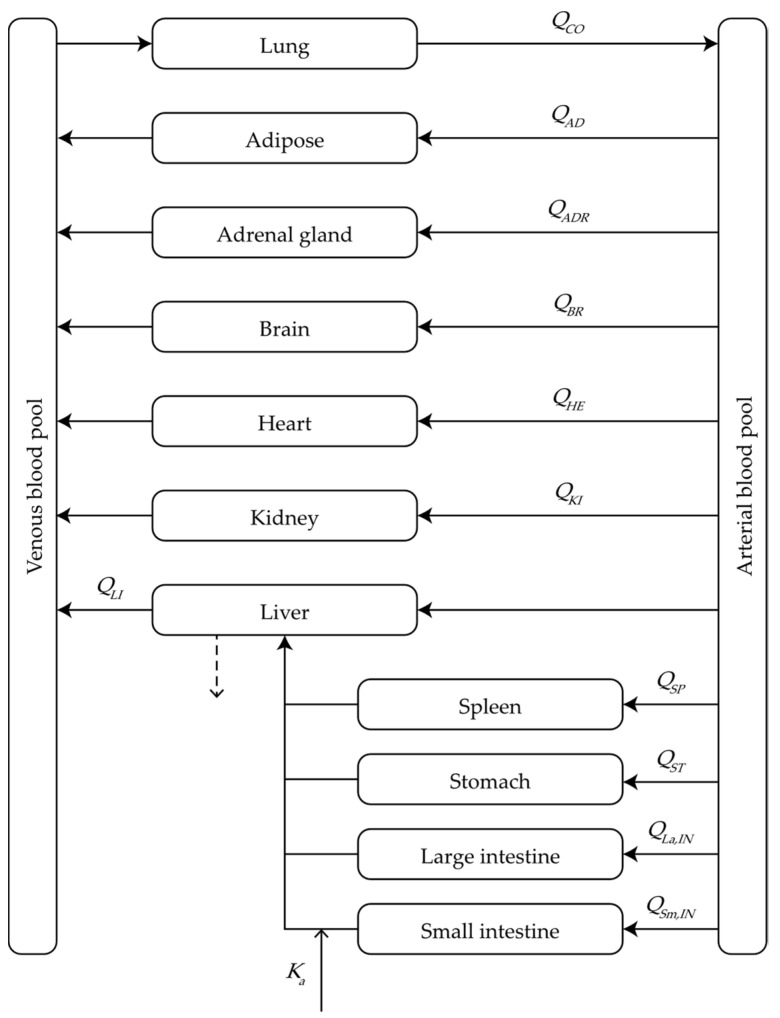
Physiological model of the pharmacokinetics of orally administered fexuprazan in humans.

The present study was the first to develop a physiological model of fexuprazan pharmacokinetics. Briefly, it consisted of 13 compartments including the arterial/venous blood pool, adipose tissue, adrenal gland, brain, heart, kidney, large and small intestines, liver, lung, spleen, and stomach (Figure 1). The anatomical volumes and blood flow rates required for the PBPK calculations were obtained from the literature [15,24] (Table 1). A detailed description of the model highlighted fexuprazan absorption, distribution, and elimination.

### 2.2. Absorption Kinetics

A first-order model was used to describe the absorption kinetics of fexuprazan. The pharmacokinetic profiles for the first dose day were obtained from a previous clinical study (Study Protocol No. NCT02757144) in a dose range of 20–80 mg/day. The fraction absorbed (*Fa*) was optimized according to the administered fexuprazan dose [16]. The first-order absorption rate ($K_{a}$) was predicted from the theoretical relationship $K_{a}=2\cdot P_{eff}/radius$, assuming that the human intestinal tract is a cylindrical tube with 1.75 cm radius. The effective permeability ($P_{eff}$) was predicted from an empirical correlation between in vitro Caco-2 permeability (nm/s) and in vivo intestinal effective permeability (μm/s), according to the following equation [25]:

(1) $$\log P_{eff}=0.4926\times \log P_{app}-0.1454$$

It was assumed that the drug absorbed from the enteric compartment was transported into the liver by blood perfusion via the portal vein.

### 2.3. Distribution Kinetics

To estimate the apparent volume of distribution of fexuprazan in humans, an allometric relationship for *V_SS_* (distribution volume at steady state) was determined from pharmacokinetic studies on rats, monkeys, and dogs. A standard moment analysis of systemic fexuprazan pharmacokinetics indicated that *V_SS_* were 20.2 L/kg for rat, 9.17 L/kg for monkey, and 12.6 L/kg for dog. A typical allometric equation ($y=a\times BW^{b}$) was used for fexuprazan to obtain the correlations between *V_SS_* and body weight (0.25 kg for rat, 4 kg for monkey, and 10 kg for dog), as follows:

(2) $$V_{SS}\;\left(L\right)=15.0\times {\left[BW\;\left(\mathrm{kg}\right)\right]}^{0.8356}\;$$

It was estimated that *V_SS_* = 7.48 L/kg for a human weighing 70 kg.

To establish the extent of tissue distribution of fexuprazan, the steady state tissue-to-plasma concentration ratios ($K_{p,SS}=AUC_{tissue}/AUC_{plasma}$) in eleven major tissues of rats were derived from a previous study (Table 2) [14,16].

Based on anatomical tissue volumes (Table 1) and $K_{p,SS}$ (Table 2), the initial *V_SS_* was calculated using the Øie-Tozer equation [26]:

(3) $$V_{SS}=V_{p}+V_{rbc}\times EP+\sum \left(V_{T,i}\times K_{p,SS}\right),$$

where $V_{p}$, $V_{rbc}$, and $V_{T}$ are the plasma, red blood cell, and tissue volumes, respectively, and EP is the erythrocyte-to-plasma partition coefficient, which is calculated as follows:

(4) EP=1+(R−1)/Hct

where Hct is the hematocrit (0.45) [27] and R is the blood-to-plasma concentration ratio (R = 0.8). Assuming that the allometric relationship determined from preclinical species is reliable for predicting human *V_SS_* (7.48 L/kg), $K_{p,scalar}$ (0.371) was multiplied by $K_{p,SS}$ for rat tissues (Table 2) to obtain $K_{p}$ applicable to human tissues. Because fexuprazan has a large extraction ratio (ER) in the liver, $K_{p,SS}$ scaled by $K_{p,scalar}$ were corrected with ER estimated from the results of an in vitro metabolite phenotyping study and preliminary simulations of additional intrinsic clearance (i.e., $CL_{u,add}$ in terms of unbound clearance) [28]. For non-eliminating organs, $K_{p,SS}$ corrected by $K_{p,scalar}$ were regarded as $K_{p}$.

For the rate of tissue distribution of fexuprazan in humans, perfusion-limited distribution was assumed based on its high in vitro permeability coefficients (16.5 ± 2.0 × 10^−6^ cm/s and 23.7 ± 4.6 × 10^−6^ cm/s in the apical to basolateral and basolateral to apical sides of Caco-2 cell systems, respectively) [16]. These values were comparable to those for propranolol, and the perfusion-limited model was also applicable for clinical pharmacokinetics of the drug [29].

### 2.4. Elimination Kinetics

According to a previous clinical study (Study Protocol No. NCT02757144 [10,22]), renal fexuprazan excretion was kinetically unimportant (i.e., Fe (fraction excreted into urine) in the range of 0.29–2.02%). In the present study, therefore, non-renal fexuprazan elimination was assumed to be primarily governed by hepatic elimination. An in vitro metabolite phenotyping study [1,16] suggested that hepatic elimination depended on the CYP3A4-mediated oxidative deamination of fexuprazan to M14 (5-(2,4-difluorophenyl)-1-([3-fluorophenyl]sulfonyl)-4-methoxy-1*H*-pyrrole-3-carboxylic acid) and the hydroxylation of fexuprazan to M11 (*N*-([5-(2,4-difluorophenyl)-1-((3-fluorophenyl)sulfonyl)-4-methoxy-1*H*-pyrrol-3-yl]methyl)-*N*-methylhydroxylamine). Based on the previous in vitro metabolism studies involving recombinant CYP enzyme activity with human liver microsomes [1], the contribution of other enzymes to the metabolic conversion of fexuprazan into M14 (8.5% by CYP2B6 and CYP2C19) and M11 (31.8% by CYP2B6 and CYP2D6) appeared to be insignificant.

The kinetic variables for fexuprazan metabolism to M14 and M11 in recombinant enzyme systems were transformed into those in human liver microsomes. Using pooled human liver microsomes (Catalog No. 452161, Batch No. 4133007, BD Gentest^TM^), testosterone 6β-hydroxylation activity (by CYP3A4) was 6100 pmol/min/mg protein. This metabolic pathway in recombinant CYP3A4 system was determined to be 200 pmol/min/pmol P450. Because CYP abundance in liver (i.e., CYP3A4 content in human liver microsome) was 0.079 nmol/mg protein [20], inter-system extrapolation factor was estimated to be 0.4 for the CYP3A4 system. Assuming a microsomal protein per gram of liver (MPPGL) value of 39.79, obtained from Simcyp V19 release 1 (Certara UK Limited, Sheffield, UK) [15], and liver weight at a liver density of unity (Table 1), *V_max_* and *K_m_* for M14 formation were estimated to be 248 nmol/min and 0.093 μM, respectively, and *V_max_* and *K_m_* for M11 formation were estimated to be 800 nmol/min and 15.95 μM, respectively. The free fraction of fexuprazan in human liver microsomes ($f_{u,mic}$) was estimated in silico to be 0.904 [21]. The intrinsic clearance of unbound hepatic fexuprazan ($CL_{u,int}$) was calculated as follows:

(5) $$CL_{u,int}=\sum \frac{V_{max}}{K_{m}\times f_{u,mic}+C_{LI}\times f_{u,LI}}+CL_{u,add}$$

where $f_{u,LI}$ is the free fraction of fexuprazan in the liver calculated by dividing the free fraction in the plasma ($f_{up}$) by the equilibrium tissue-to-plasma partition coefficient for the liver ($K_{p,LI}$). $CL_{u,add}$ (the unbound additional intrinsic clearance) consists of biliary excretion and miscellaneous hepatic elimination pathways other than metabolic M14 and M11 formation, which was optimized depending on the administered fexuprazan dose.

### 2.5. PBPK Calculations

For the fexuprazan absorption kinetics, the differential equation for the amount of drug in the enteral compartment is:

(6) $$\frac{dX_{a}}{dt}=-K_{a}\times X_{a}$$

where $X_{a}$ is the amount of drug remaining in the enteral compartment and $K_{a}$ is the first-order absorption rate constant. The initial amount of fexuprazan in the absorption compartment was the product of *Fa* and the administered fexuprazan dose. Oral bioavailability (F) of fexuprazan in humans was estimated using the following relationship:

(7) $$F=F_{a}\times F_{g}\times F_{h}$$

(8) $$F_{h}=\frac{Q_{LI}R}{Q_{LI}R+f_{up}CL_{u,int}}$$

where $F_{g}$ is the fraction escaping from gut wall extraction (e.g., metabolism), $F_{h}$ is the hepatic availability, and $Q_{LI}$ is the hepatic blood flow. In this calculation, $F_{g}$ was assumed to be unity for fexuprazan.

Assuming a perfusion-limited fexuprazan distribution rate, the differential equation for all tissues other than the liver was:

(9) $$V_{T}\frac{dC_{T}}{dt}=Q_{T}\cdot \left(C_{art}-\frac{C_{T}\times R}{K_{p}}\right)$$

where $V_{T}$ is the volume of tissue compartment, $C_{T}$ and $C_{art}$ are the drug concentrations in the tissue and arterial blood compartments, respectively, $Q_{T}$ is the blood flow to the tissue, R is the blood-to-plasma concentration ratio, and $K_{p}$ is the equilibrium tissue-to-plasma concentration ratio.

For the liver compartment:

(10) $$\begin{array}{c} V_{LI}\frac{dC_{LI}}{dt}=K_{a}\times X_{a}+(Q_{LI}-Q_{ST}-Q_{SP}-Q_{Sm,IN}-Q_{La,IN})\cdot C_{art}+Q_{ST}\frac{C_{ST}\times R}{K_{p,ST}}+Q_{SP}\frac{C_{SP}\times R}{K_{p,SP}}\\ \;\;\;\;\;\;\;+Q_{Sm,IN}\frac{C_{Sm,IN}\times R}{K_{p,Sm,IN}}+Q_{La,IN}\frac{C_{La,IN}\times R}{K_{p,La,IN}}-Q_{LI}\frac{C_{LI}\times R}{K_{p,LI}}-CL_{u,int}\frac{f_{up}}{K_{p,LI}}C_{LI} \end{array}$$

where $V_{LI}$ is the liver volume; $C_{LI}$, $C_{ST}$, $C_{SP}$, $C_{Sm,IN}$, and $C_{La,IN}$ are the drug concentrations in the liver, stomach, spleen, and small and large intestines, respectively; $Q_{LI}$, $Q_{ST}$, $Q_{SP}$ $Q_{Sm,IN}$, and $Q_{La,IN}$ are the blood flow to liver, stomach, spleen, and small and large intestines, respectively; $K_{p,LI}$, $K_{p,ST}$, $K_{p,SP}$, $K_{p,Sm,IN}$, and $K_{p,La,IN}$ are the equilibrium tissue-to-plasma concentration ratios for the liver, stomach, spleen, and small and large intestines, respectively; and $CL_{u,int}$ is the intrinsic drug molecule clearance in the liver compartment.

For the venous blood compartment:

(11) $$\begin{array}{c} V_{ven}\frac{dC_{ven}}{dt}=Q_{ADR}\frac{C_{ADR}\times R}{K_{p,ADR}}+Q_{AD}\frac{C_{AD}\times R}{K_{p,AD}}+Q_{BR}\frac{C_{BR}\times R}{K_{p,BR}}+Q_{HE}\frac{C_{HE}\times R}{K_{p,HE}}+Q_{KI}\frac{C_{KI}\times R}{K_{p,KI}}+Q_{LI}\frac{C_{LI}\times R}{K_{p,LI}}\\ \;\;\;\;\;\;+Q_{RE}\times C_{art}-Q_{CO}\times C_{ven} \end{array}$$

where $V_{ven}$ is the venous blood volume; $C_{ADR}$, $C_{AD}$, $C_{BR}$, $C_{HE}$, $C_{KI}$, and $C_{ven}$ are the drug concentrations in the adrenal gland, adipose, brain, heart, kidney, and venous blood compartment, respectively; $Q_{ADR}$, $Q_{AD}$, $Q_{BR}$, $Q_{HE}$, $Q_{KI}$, and $Q_{RE}$ are the blood flows to the adrenal gland, adipose, brain, heart, and kidney, and the residual blood flow, respectively; $Q_{CO}$ is the cardiac output; and $K_{p,ADR}$, $K_{p,AD}$, $K_{p,BR}$, $K_{p,HE}$, and $K_{p,KI}$ are the equilibrium tissue-to-plasma concentration ratios of the adrenal gland, adipose, brain, heart, and kidney, respectively.

For the lung compartment:

(12) $$V_{LU}\frac{dC_{LU}}{dt}=Q_{CO}\times \left(C_{ven}-\frac{C_{LU}\times R}{K_{p,LU}}\right)$$

where $V_{LU}$ is the lung volume, $C_{LU}$ is the drug concentration in the lung, and $K_{p,LU}$ is the equilibrium tissue-to-plasma concentration ratio for the lung.

For the arterial blood compartment:

(13) $$V_{art}\frac{dC_{art}}{dt}=Q_{CO}\times \left(\frac{C_{LU}\times R}{K_{p,LU}}-C_{art}\right)$$

where $V_{art}$ is the arterial blood volume.

All the input parameters for PBPK modeling of fexuprazan in man were summarized in Table 3. *Fa*, $K_{a}$, and $CL_{u,add}$ were optimized with Winnonlin Professional 5.0.1 (Pharsight Corp., Mountain View, CA, USA). Numerical simulations of the PBPK models were performed with Berkeley Madonna v. 10.1.3 (Berkeley Madonna, Inc., Albany, CA, USA). In the present study, the fourth order of the Runge–Kutta method was used for numerical integration. GraphPad Prism v. 9.0.0 (GraphPad Software, San Diego, CA, USA) was used to visualize the simulation.

### 2.6. Modelling Strategies

During model refinement, clinical data [16,22] from a multiple ascending dose (MAD) study on fexuprazan (Study Protocol No. NCT02757144 [10,22]) for the range of 20–80 mg/day were used. The relevant parameters in the PBPK models were optimized to the pharmacokinetic data for fexuprazan at the first dose day (i.e., before the second dosing). The proposed PBPK model was validated by comparing AUC_last_ (i.e., the area under the curve from time 0 to the last sampling time) and C_max_ (the maximum concentrations) from the model simulations against those from the clinical data of the MAD study on fexuprazan at the seventh day of dose (Study Protocol No. NCT02757144 [10,22]) and other clinical datasets for Japanese, Caucasian, and Korean populations (Study Protocol No. NCT03574415 [1,23]). In the present study, the fold differences of the resulting AUC ratios (AUC_pred_:AUC_obs_) and C_max_ ratios (C_max,pred_:C_max,obs_) within a factor of two were considered adequate for model performance prediction.

### 2.7. Statistical Analysis

Means between/among groups were compared with unpaired *t*-tests or one-way ANOVA, followed by the Tukey’s post hoc test. In the present study, data were expressed as means ± SD. *p* < 0.05 denoted statistical significance.

## 3. Results

### 3.1. Establishment and Optimization of the PBPK Model for Fexuprazan in Humans

**Figure 2:**
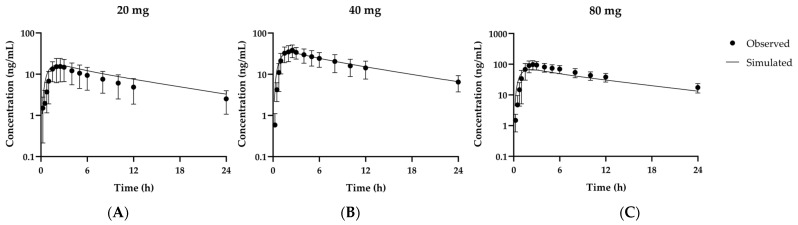
Observed and simulated time-concentration profiles for fexuprazan at the first doses of 20 mg (**A**), 40 mg (**B**), and 80 mg (**C**) in humans. Solid lines represent optimized simulations or model fitting. Closed circles (●) represent observed data [10,22]. Data are means ± SD for eight healthy volunteers.

Kinetic parameters for absorption (*Fa*) and elimination ($CL_{u,add}$) of fexuprazan were obtained by fitting the plasma concentration profiles of 24 individuals orally administered 20 mg, 40 mg, or 80 mg fexuprazan (i.e., profiles for eight volunteers per dose) [10,22]. For each dose, the *Fa* estimates were 0.627 ± 0.298, 0.767 ± 0.267, and 0.890 ± 0.344, and $CL_{u,add}$ estimates were 15.8 ± 7.21 L/min, 13.9 ± 8.16 L/min, and 8.95 ± 5.38 L/min. Because neither *Fa* nor $CL_{u,add}$ significantly differed among doses based on one-way ANOVA, linear pharmacokinetics was assumed for fexuprazan absorption and hepatic clearance at doses in the range of 20–80 mg. The average *Fa* and $CL_{u,add}$ of 0.761 and 12.9 L/min for the 24 volunteers were used to predict fexuprazan pharmacokinetics in other clinical datasets and to validate the model. Fexuprazan model simulations for optimization are shown in Figure 2. The AUC_last_ and C_max_ ratios were in the range of 0.672–1.32 (Table 4). Using the established model, the absolute bioavailability could be estimated for orally administered fexuprazan. Briefly, the time-concentration profiles could be simulated using the validated PBPK model after intravenous or oral administration of 20, 40, or 80 mg fexuprazan in humans, and AUC ratio (i.e., AUC_PO_/AUC_IV_) was estimated for each dose. AUC was calculated based on the time-concentration curve, and the bioavailability could be calculated as 38.4%, 38.4%, and 38.6% for 20, 40, and 80 mg doses, respectively. 

### 3.2. Validation of the Fexuprazan PBPK Model for Humans

**Figure 3:**
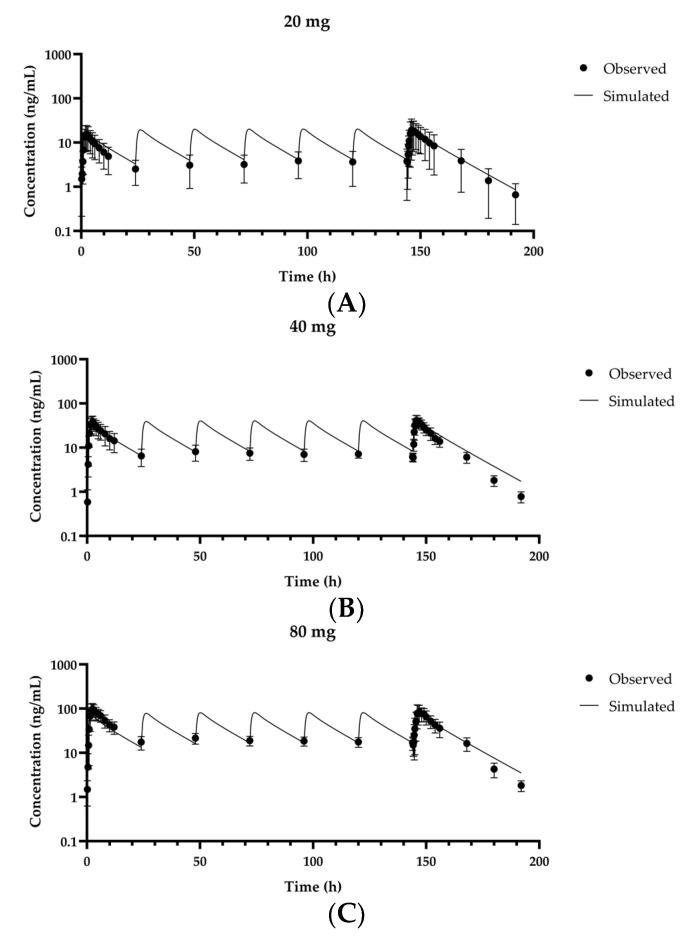
Observed and predicted fexuprazan concentrations at repeated doses of 20 mg/day (**A**), 40 mg/day (**B**), and 80 mg/day (**C**) administered to humans for 7 days. Solid lines represent simulated results of optimized model or model prediction. Closed circles (●) represent observed data [10,22]. Data are means ± SD for eight healthy volunteers.

In the present study, we used in silico, in vitro, and in vivo data to propose PBPK models for fexuprazan orally administered to humans. The PBPK model was optimized using first dose day data from the MAD study and validated with dose day 7 data from the same study [10,22] as well as clinical data for various ethnic groups [1,23]. The AUC_last_ and C_max_ ratios were in the ranges of 0.880–1.06 and 0.861–0.972, respectively (Table 4). When the plasma fexuparazan concentrations for dose days 1–7 of the MAD study were plotted, the model simulations reasonably captured the Day 1 data along with the trough concentrations at Day 7 after multiple dosing (Figure 3).

**Figure 4:**
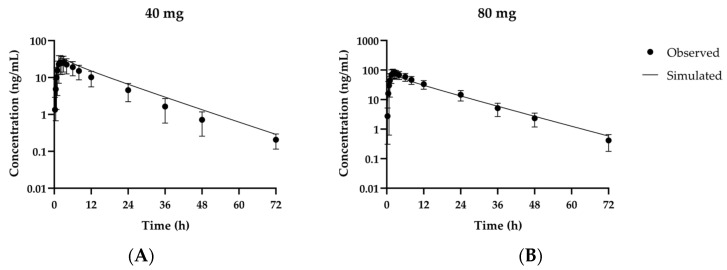
Observed and simulated fexuprazan time-concentration profiles at first doses of 40 mg (**A**) and 80 mg (**B**) in Korean, Caucasian, and Japanese populations. Solid lines represent results of optimized simulation or model fitting. Closed circles (●) represent observed data [1,23]. Data are as means ± SD for 24 volunteers including all Korean, Caucasian, and Japanese populations.

**Figure 5:**
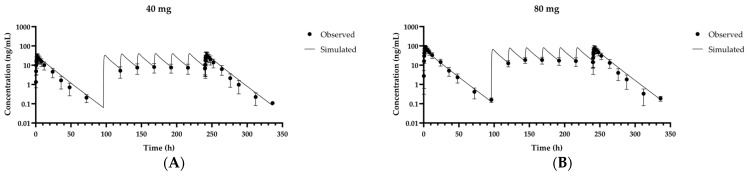
Observed and predicted fexuprazan concentration profiles at repeated doses of 40 mg/day (**A**) and 80 mg/day (**B**) on days 1, 5, 6, 7, 8, 9, 10, and 11 in Korean, Caucasian, and Japanese populations. Solid lines represent simulated results of optimized model or model prediction. Closed circles (●) represent observed data [1,23]. Data are means ± SD for 24 volunteers including all Korean, Caucasian, and Japanese populations.

Another Phase 1 study was conducted on Japanese, Caucasian, and Korean subjects [1,23]. The clinical dataset comprised the first day after single 40 mg and 80 mg doses in all three populations. The proposed model reasonably predicted systemic pharmacokinetics for 48 h after the first and last fexuprazan doses, with the AUC_last_ and C_max_ ratios in the ranges of 0.905–1.42 and 0.770–1.15, respectively (Table 4). Steady state pharmacokinetics after the eighth dose administered to all three populations was adequately predicted with AUC_last_ and C_max_ ratios in the ranges of 1.23–1.32 and 1.03–1.14, respectively. These results were consistent with visual inspections of the simulated concentrations. The simulated and observed time-concentration profiles fit well for the first dose day (Figure 4) and subsequent fexuprazan administrations over 9 days (Figure 5).

## 4. Discussion

Pharmacokinetic modeling quantitatively clarifies the in vivo kinetic behavior of new compounds. Unlike conventional compartmental analyses, PBPK models comprise numerous system-specific (physiological/anatomical) and drug-specific (physicochemical) parameters that can elucidate and compare pharmacokinetics across preclinical and clinical species. Depending on the quantity and quality of available data, pharmacokinetic models may be established by combining ‘bottom-up’ or ‘top-down’ approaches [30] to determine the model parameter(s). Despite insufficient in silico/in vitro/in vivo data for the clinical pharmacokinetics of fexuprazan, the present PBPK model adequately described and predicted numerous clinical datasets from various populations at clinically relevant doses. This model may also be expanded to predict fexuprazan pharmacokinetics under different clinical settings such as specific populations and combinations of fexuprazan with other drugs. However, further studies might have to be performed for mechanistic establishment of the kinetic parameters not identified via in vitro/in silico approaches such as *Fa* and $CL_{u,add}$.

Previously, other metabolites such as M7 (1-(5-[2,4-difluorophenyl]-1-[(3-fluorophenyl)sulfonyl]-4-methoxy-1*H*-pyrrol-3-yl)-N-methylmethanamine) were not detectable in human plasma 7 days after oral administration of 160 mg/day. In the present study, therefore, we optimized $CL_{u,add}$, which represents miscellaneous hepatic elimination pathways other than metabolic M14 and M11 formation. The fractional contribution by the various metabolic pathways (%fm) could be estimated using the terms in Equation (5). The %fm values were determined to be 18.5%, 0.349%, and 81.1% for the formations of M14 and M11 (primarily by CYP3A4), and $CL_{u,add}$, respectively. The previously approved P-CAB vonoprazan was radiolabeled and administered to rats and 88% of its total radioactivity was recovered from the bile [31]. A similar finding was recorded for radiolabeled fexuprazan in rats (88% recovered up to 48 h) (NCE001-4225-REP-002). In the case of vonoprazan, however, the parent drug exhibited negligible biliary excretion in bile duct-cannulated rats, and there was minimal fecal recovery of the parent drug in dogs [31]. These findings were consistent with the empirical MW cutoff for kinetically insignificant biliary excretion, namely, <10% for drugs with MW < 475 Da [32]. Moreover, metabolite profiles were not fully identified for vonoprazan excreted in bile as there remained 53.8% miscellaneous radioactivity in rat bile. One of our preclinical studies using rats disclosed that various metabolites of fexuprazan were identified in bile samples (data not shown). Hence, further studies should be conducted to identify the kinetic component of $CL_{u,add}$ for P-CAB drugs.

The sufficient solubility and permeability of fexuprazan [16] is consistent with our modeling result of a high *Fa* (0.761). The first-order rate constant ($K_{a}$) of fexuprazan calculated from in vitro Caco-2 permeability (Equation (1)) [25] adequately predicted the absorptive phase of in vivo pharmacokinetics. At the optimized *Fa* of 0.761, absolute fexuprazan bioavailability in humans is estimated to be 38.4–38.6%, which is comparable to preclinical data (3.89–50.6%). Assuming unlimited fexuprazan solubility and permeability in the small intestine, observed differences in fexuprazan bioavailability among species could be partially explained by interspecies differences in the first-pass effect in the gut wall and/or liver.

For the distribution of fexuprazan to human tissues, a correction factor of 0.371 ($K_{p}$ scalar) was required for consistency with the allometric relationship on *V_SS_*. Assuming the absence of active transport across the membrane, $K_{p}$ might depend mainly on drug binding to plasma/tissue constituents. The free fraction of vonoprazan in the plasma (fup) was higher in rats than in humans (30.5–32.7% and 13.5–14.8%, respectively). Therefore, the fexuprazan distribution volume may be comparatively higher in rats. However, plasma protein binding of fexuprazan did not significantly differ across species (93.1–93.9%, 92.7–92.8%, 88.3–91.0%, and 92.8–94.3% for rats, beagles, cynomolgus monkeys, and humans, respectively). Considering that fexuprazan may be bound to more tissue constituents than plasma proteins (*V_SS_* = 9.17–20.2 L/kg in preclinical species), a $K_{p}$ scalar of 0.371 was required, likely because of differential fexuprazan tissue binding across species.

Our proposed PBPK model revealed that AUC_ratio_ and C_max,ratio_ were in the ranges of 0.880–1.42 and 0.770–1.15, respectively (Table 4). These values were consistent with our visual inspections (Figure 3, Figure 4 and Figure 5). Validation of the proposed PBPK model indicated that its structure and each parameter were appropriate for the prediction of plasma concentration profiles following oral administration of fexuprazan in humans in the therapeutic range of 20–80 mg/day within the cutoff criterion of a factor of two.

In the proposed PBPK model, fexuprazan absorption was assumed to follow first-order kinetics. *Fa* was estimated by fitting the observed data while considering interspecies differences in fexuprazan bioavailability. Though the PBPK model predictions reasonably captured clinical data, the apparent absorption kinetic parameters (*Fa* and $K_{a}$) calculated here would be influenced by animal physiology (e.g., relative GI tract length, pH, and water content) and drug physicochemical properties (e.g., relative solubility over a pH range and first-pass metabolism). Variability in the foregoing factors could lead to divergent bioavailability values [33]. In preclinical animals, there was species-dependent oral bioavailability in the range of 3.89–50.6%. Thus, mechanistic absorption models might be used to analyze factors governing overall absorption, such as ADAM (advanced dissolution, absorption, and metabolism) and ACAT (advanced compartmental absorption and transit) that incorporate intestinal metabolism and active transport [34].

The PBPK model proposed herein could serve to predict or describe potential interactions between fexuprazan and other co-administered drugs. The P-CAB drug tegoprazan has been prescribed for *H. pylori* eradication in South Korea [8,35], and thus fexuprazan could also be co-administered with amoxicillin and clarithromycin to treat *H. pylori* infection. However, clarithromycin is a CYP3A4 inhibitor and could, therefore, interact with fexuprazan, a CYP3A substrate. The PBPK model established here could quantitatively evaluate the clinical significance of DDI risk in ongoing fexuprazan development and optimization. It might also be expanded to analyze fexuprazan pharmacokinetics for elderly and pediatric populations if adequate physiological information can be incorporated.

In conclusion, a PBPK model for orally administered fexuprazan in humans was developed and validated here by integrating in vitro, in vivo, and in silico data. The model may be used to predict potential DDIs as well as pharmacokinetic profiles in various clinical situations, including elderly and pediatric populations as well as patients with renal or hepatic impairment.

## Figures and Tables

**Table 1:** Summary of physiological input parameters used for the PBPK model. The cardiac output for a representative human of 70 kg body weight was set to be 5200 mL/min in this study [15,24].

Tissue	Volume (mL)	Blood Flow (mL/min)
Adipose	15,000	270
Adrenal gland	14	15.6
Brain	1400	593
Heart	329	208
Kidney	308	910
Large Intestine	371	208
Liver	1800	1326
Lung	532	5200
Small Intestine	520	520
Spleen	182	104
Stomach	147	52
Venous blood	3470	
Arterial blood	1730	

**Table 2:** Summary of steady state tissue-to-plasma concentration ratios of fexuprazan in 11 major tissues of rats.

Tissue	*K_p,SS_*
Adipose	11.7
Adrenal gland	56.1
Brain	3.55
Heart	12.4
Kidney	44.2
Large Intestine	110
Liver	417
Lung	236
Small Intestine	637
Spleen	47.9
Stomach	519

**Table 3:** Input parameters for PBPK modeling of fexuprazan in humans.

Category	Parameter (unit)	Value	Comments
Physicochemical Properties and Blood Binding	Compound type	Base	
	pKa	9.04	Determined [16]
	logP	2.38	
	*f_up_*	0.0645	
	B/P ratio (R)	0.8	
Absorption	*K_a_* (min^−1^)	0.0606	Predicted (See text)
	*Fa*	0.761	Optimized (See text)
Distribution (*K_p_*) *	Adrenal gland	20.8	Corrected by *K_p,scalar_* (See text)
	Adipose	4.32	
	Brain	1.32	
	Heart	4.60	
	Kidney	16.4	
	Liver	303	
	Lung	87.6	
	Large Intestine	40.8	
	Small Intestine	124	
	Spleen	17.8	
	Stomach	193	
Elimination	$f_{u,mic}$	0.904	Predicted (See text)
	$CL_{u,add}$ (L/min)	12.9	Optimized (See text)
M14 Formation by CYP3A4	$V_{max}$ (nmol/min)	248	Determined [16]
	$K_{m}$ (μM)	0.093	
M11 Formation by CYP3A4	$V_{max}$ (nmol/min)	800	Determined [16]
	$K_{m}$ (μM)	15.95	

* Tissue-to-plasma partition coefficients ($K_{p}$) were corrected from the values of rat tissues (Table 2).

**Table 4:** Summary of AUC_last_ (ng·min/L) and C_max_ (ng/mL) ratios of fexuprazan in the two clinical trials and simulations.

Dose	AUC_obs_	AUC_pred_	AUC_ratio_	C_max,obs_	C_max,pred_	C_max ratio_
*Training set (1st day MAD)* ^1^						
20 mg	9020	11,900	1.32	16.3	16.6	1.02
40 mg	23,700	23,900	1.01	40.4	33.2	0.822
80 mg	62,400	48,000	0.770	99.1	66.6	0.672
*Validation set (7th day MAD)* ^1^						
20 mg/day	16,300	14,900	0.916	20.8	20.2	0.972
40 mg/day	28,300	30,000	1.06	43.2	40.4	0.935
80 mg/day	68,700	60,400	0.880	94.4	81.2	0.861
*Validation set (1st dose)* ^2^						
40 mg	21,000	29,800	1.42	28.8	33.2	1.15
80 mg	66,300	60,000	0.905	86.4	66.6	0.770
*Validation set (8th dose)* ^2^						
40 mg/day	28,300	37,500	1.32	35.5	40.4	1.14
80 mg/day	61,800	75,700	1.23	78.9	81.2	1.03

^1^ Observed data from the registered clinical trial in healthy volunteers (registered at ClinicalTrials.gov as NCT02757144 [10,22]). The observed value is the average value of the pharmacokinetic parameters obtained from the data of eight people. ^2^ Observed data from the registered clinical trial among Korean, Caucasian, and Japanese (registered at ClinicalTrials.gov as NCT03574415 [1,23]). The observed value is the average value of the pharmacokinetic parameters obtained from the data of 24 people.

## Data Availability

The cited clinical trial data were already published by Sunwoo et al. (NCT02757144) [10] and Hwang et al. (NCT03574415) [1].

## References

[1] Hwang J.G., Jeon I., Park S.A., Lee A., Yu K.-S., Jang I.-J., Lee S. (2020). Pharmacodynamics and Pharmacokinetics of DWP14012 (Fexuprazan) in Healthy Subjects with Different Ethnicities. Aliment. Pharmacol. Ther..

[2] Chey W.D., Leontiadis G.I., Howden C.W., Moss S.F. (2017). ACG Clinical Guideline: Treatment of Helicobacter pylori Infection. Am. J. Gastroenterol..

[3] Kahrilas P.J. (2008). Gastroesophageal Reflux Disease. N. Engl. J. Med..

[4] Kim J.-H., Kim B.J., Kim S.W., Kim S.E., Kim Y.S., Sung H.Y., Oh T.-H., Jeong I.D., Park M.I. (2014). Current Issues on Gastroesophageal Reflux Disease. Korean J. Gastroenterol..

[5] Rawla P., Sunkara T., Ofosu A., Gaduputi V. (2018). Potassium-Competitive Acid Blockers—Are They the Next Generation of Proton Pump Inhibitors?. World J. Gastrointest. Pharmacol. Ther..

[6] Hunt R.H., Scarpignato C. (2018). Potent Acid Suppression with PPIs and P-CABs: What’s New?. Curr. Treat. Options Gastroenterol..

[7] Sugano K. (2018). Vonoprazan Fumarate, a Novel Potassium-Competitive Acid Blocker, in the Management of Gastroesophageal Reflux Disease: Safety and Clinical Evidence to Date. Ther. Adv. Gastroenterol..

[8] Takahashi N., Take Y. (2018). Tegoprazan, a Novel Potassium-Competitive Acid Blocker to Control Gastric Acid Secretion and Motility. J. Pharmacol. Exp. Ther..

[9] Sunwoo J., Ji S.C., Oh J., Ban M.S., Nam J.Y., Kim B., Song G.S., Yu K.-S., Jang I.-J., Lee S. (2020). Pharmacodynamics of Tegoprazan and Revaprazan after Single and Multiple Oral Doses in Healthy Subjects. Aliment. Pharmacol. Ther..

[10] Sunwoo J., Oh J., Moon S.J., Ji S.C., Lee S.H., Yu K.S., Kim H.S., Lee A., Jang I.J. (2018). Safety, Tolerability, Pharmacodynamics and Pharmacokinetics of DWP14012, a Novel Potassium-Competitive Acid Blocker, in Healthy Male Subjects. Aliment. Pharmacol. Ther..

[11] Galetin A., Burt H., Gibbons L., Houston J.B. (2006). Prediction of Time-Dependent CYP3A4 Drug-Drug Interactions: Impact of Enzyme Degradation, Parallel Elimination Pathways, and Intestinal Inhibition. Drug Metab. Dispos..

[12] Pal D., Mitra A.K. (2006). MDR- and CYP3A4-Mediated Drug–Drug Interactions. J. Neuroimmune Pharmacol..

[13] Graham D.Y., Dore M.P. (2018). Update on the Use of Vonoprazan: A Competitive Acid Blocker. Gastroenterology.

[14] Yim C.-S., Jeong Y.-S., Lee S.-Y., Pyeon W., Ryu H.-M., Lee J.-H., Lee K.-R., Maeng H.-J., Chung S.-J. (2017). Specific Inhibition of the Distribution of Lobeglitazone to the Liver by Atorvastatin in Rats: Evidence for a Rat Organic Anion Transporting Polypeptide 1B2–Mediated Interaction in Hepatic Transport. Drug Metab. Dispos..

[15] Jamei M., Marciniak S., Feng K., Barnett A., Tucker G., Rostami-Hodjegan A. (2009). The Simcyp^®^ Population-Based ADME Simulator. Expert Opin. Drug Metab. Toxicol..

[16] (2018). Investigator’s Brochure DWP14012.

[17] Zheng Y., Benet L.Z., Okochi H., Chen X. (2015). pH Dependent but not P-gp Dependent Bidirectional Transport Study of S-propranolol: The Importance of Passive Diffusion. Pharm. Res..

[18] Haltner-Ukomadu E., Gureyeva S., Burmaka O., Goy A., Mueller L., Kostyuk G., Margitich V. (2018). In Vitro Bioavailability Study of an Antiviral Compound Enisamium Iodide. Sci. Pharm..

[19] Wang Y., Cao J., Wang X., Zeng S. (2010). Stereoselective Transport and Uptake of Propranolol across Human Intestinal Caco-2 Cell Monolayers. Chirality.

[20] Rostami-Hodjegan A., Tucker G.T. (2007). Simulation and Prediction of in Vivo Drug Metabolism in Human Populations From in Vitro Data. Nat. Rev. Drug Discov..

[21] Turner D.B., Rostami-Hodjegan A., Tucker G.T., Yeo K.R. (2006). Prediction of Non-Specific Hepatic Microsomal Binding from Readily Available Physicochemical Properties Available Physicochemical Properties. Drug Metab. Rev..

[22] (2017). ClinicalTrials.gov (NCT02757144): Safety, Tolerability, Pharmacokinetics and Pharmacodynamics of DWP14012 after Oral Administration in Healthy Male Volunteers.

[23] (2019). ClinicalTrials.gov (NCT03574415): Safety, Tolerability, Pharmacokinetics and Pharmacodynamics of DWP14012 after Oral Administration in Healthy Japanese, Caucasian and Korean.

[24] Brown R.P., Delp M.D., Lindstedt S.L., Rhomberg L.R., Beliles R.P. (1997). Physiological Parameter Values for Physiologically Based Pharmacokinetic Models. Toxicol. Ind. Health.

[25] Gertz M., Harrison A., Houston J.B., Galetin A. (2010). Prediction of Human Intestinal First-Pass Metabolism of 25 CYP3A Substrates from In Vitro Clearance and Permeability Data. Drug Metab. Dispos..

[26] Øie S., Tozer T.N. (1979). Effect of Altered Plasma Protein Binding on Apparent Volume of Distribution. J. Pharm. Sci..

[27] Billett H.H., Walker H.K., Hall W.D., Hurst J.W. (1990). Clinical Methods: The History, Physical, and Laboratory Examinations.

[28] Berezhkovskiy L.M. (2010). A Valid Equation for the Well-Stirred Perfusion Limited Physiologically Based Pharmacokinetic Model that Consistently Accounts for the Blood–Tissue Drug Distribution in the Organ and the Corresponding Valid Equation for the Steady State Volume of Distribution. J. Pharm. Sci..

[29] Kiriyama A., Honbo A., Iga K. (2008). Analysis of Hepatic Metabolism Affecting Pharmacokinetics of Propranolol in Humans. Int. J. Pharm..

[30] Tsamandouras N., Rostami-Hodjegan A., Aarons L. (2015). Combining the ‘Bottom up’ and ‘Top down’ Approaches in Pharmacokinetic Modelling: Fitting PBPK Models to Observed Clinical Data. Br. J. Clin. Pharmacol..

[31] Kogame A., Takeuchi T., Nonaka M., Yamasaki H., Kawaguchi N., Bernards A., Tagawa Y., Morohashi A., Kondo T., Moriwaki T. (2017). Disposition and Metabolism of TAK-438 (Vonoprazan Fumarate), a Novel Potassium-Competitive Acid Blocker, in Rats and Dogs. Xenobiotica.

[32] Yang X., Gandhi Y.A., Duignan D.B., Morris M.E. (2009). Prediction of Biliary Excretion in Rats and Humans Using Molecular Weight and Quantitative Structure-Pharmacokinetic Relationships. AAPS J..

[33] Hatton G.B., Yadav V., Basit A.W., Merchant H.A. (2015). Animal Farm: Considerations in Animal Gastrointestinal Physiology and Relevance to Drug Delivery in Humans. J. Pharm. Sci..

[34] Huang W., Lee S.L., Yu L.X. (2009). Mechanistic Approaches to Predicting Oral Drug Absorption. AAPS J..

[35] (2020). HK inno.N Corp. K-CAB. Package Insert. https://nedrug.mfds.go.kr/pbp/CCBBB01/getItemDetail?itemSeq=201802815.

